# Delayed Diagnosis and Multidisciplinary Management of a Compound Odontoma With Associated Dental Anomalies: A Rare Case Report

**DOI:** 10.1155/crid/5578256

**Published:** 2025-12-19

**Authors:** Hussam Bajunaid, Yassin Hemoudi, Saeed Alqalaleef, Rakan Alshihri, Yousef Ezzat, Hisham Komo, Rayan Sharka, Hassan Abed

**Affiliations:** ^1^ Dental and Oral Surgery Department, Royal Commission Medical Center, Yanbu, Saudi Arabia, rcymc.med.sa; ^2^ Department of Oral and Maxillofacial Surgery, King Abdulaziz University Dental Hospital, Jeddah, Saudi Arabia; ^3^ Department of Oral and Maxillofacial Surgery, Faculty of Dental Medicine, Umm Al-Qura University, Makkah, Saudi Arabia, uqu.edu.sa; ^4^ Department of Basic and Clinical Oral Science, Faculty of Dental Medicine, Umm Al-Qura University, Makkah, Saudi Arabia, uqu.edu.sa

**Keywords:** dental anomaly, maxillofacial surgery, odontogenic tumors, orthodontic traction, surgical exposure, tooth eruption disorders

## Abstract

Hyperdontia is a developmental anomaly that can disrupt normal tooth eruption and occlusal harmony. Compound odontomas, although benign and often asymptomatic, may cause delayed eruption and other complications requiring surgical management. This case report details the treatment of a 22‐year‐old female presenting with failure of eruption of the mandibular left permanent canine. Clinical and radiographic evaluation revealed a retained deciduous canine, a compound odontoma, and a horizontally impacted supernumerary tooth. Surgical intervention under local anesthesia involved extraction of the retained deciduous and supernumerary teeth, complete removal of the odontoma, and surgical exposure of the impacted canine with placement of an orthodontic traction chain to facilitate eruption. Postoperative recovery was uneventful, with satisfactory soft tissue healing and partial eruption of the permanent canine observed at the 3‐month follow‐up. This case emphasizes the importance of early diagnosis and timely management of odontogenic anomalies to prevent functional and esthetic complications. Coordinated surgical and orthodontic intervention can significantly improve prognosis and treatment outcomes.

## 1. Introduction

Dental anomalies represent deviations from normal development that affect tooth number, shape, size, or position due to disturbances during odontogenesis [1]. Among these, hyperdontia, or the presence of supernumerary teeth, is a well‐recognized anomaly that can disrupt normal eruption patterns and occlusal harmony. It is frequently associated with complications such as impaction of permanent teeth, malocclusion, and overretention of primary teeth, which may predispose to further anomalies including odontomas [2].

Compound odontoma is an odontogenic hamartoma composed of enamel, dentin, cementum, and pulp arranged in a tooth‐like pattern [1] (Table 1). Although benign and often asymptomatic, delayed diagnosis can result in significant complications such as tooth impaction, malocclusion, and cystic changes [3, 4]. In rare cases, odontomas may coexist with other pathologies, such as dentigerous cysts, further complicating treatment. Aldelaimi and Khalil [5] reported a case where a compound odontoma was associated with a dentigerous cyst, causing mechanical obstruction of eruption and requiring surgical removal of the impacted tooth along with cyst enucleation. This highlights the importance of early detection and comprehensive treatment planning to prevent functional and esthetic compromise.

**Table 1:** Key features of compound odontoma.

**Feature**	**Description**
Type	Benign odontogenic tumor/hamartoma
Tissue composition	Enamel, dentin, pulp, cementum
Appearance	Multiple small tooth‐like structures (denticles)
Location	More common in anterior maxilla (upper front jaw)
Age group affected	Usually found in children and adolescents (often < 20 years old)
Symptoms	Usually asymptomatic but may cause delayed tooth eruption or swelling
Radiographic appearance	Radiopaque (white) area with multiple small tooth‐like structures, surrounded by a radiolucent (dark) halo
Treatment	Surgical excision (simple and curative)
Prognosis	Excellent; rare recurrence

Impaction is not limited to permanent teeth; although rare, deciduous teeth can also fail to erupt. A previous study has described a case of complete impaction of a mandibular second deciduous molar in a 9‐year‐old patient, which led to delayed eruption of the successor premolar. Such cases emphasize that impaction in primary dentition, though uncommon, can cause serious developmental disturbances, including ectopic eruption and malocclusion. Proper history, clinical examination, and timely surgical intervention are essential to avoid long‐term complications [6].

Management strategies for odontomas and impactions vary depending on lesion size, location, and associated anomalies. While conservative monitoring may be appropriate for small, asymptomatic lesions, surgical excision is often indicated when odontomas impede eruption or are associated with cystic changes. In many cases, combined surgical and orthodontic intervention is necessary to restore normal occlusion and esthetics [5].

The present case report describes a unique scenario involving delayed eruption of a mandibular canine due to a retained deciduous tooth, a compound odontoma, and a horizontally impacted supernumerary tooth. This case underscores the critical role of early clinical and radiographic assessment in preventing complex treatment needs and optimizing patient outcomes.

## 2. Case Presentation

### 2.1. Patient Information and Clinical Findings

A healthy 22‐year‐old female patient presented to the dental clinic with congenital absence of the lower left permanent canine (Tooth #33), accompanied by retention of the corresponding deciduous tooth (Tooth #73). The patient reported no discomfort, pain, or functional limitations. Clinical examination revealed no signs of swelling, inflammation, or infection in the region of the missing tooth. The patient′s mother reported no history of facial trauma or developmental disturbances in the mandibular anterior region. Additionally, her medical history, including pregnancy and childbirth, was unremarkable.

### 2.2. Diagnostic Assessment

A panoramic radiograph was obtained, revealing multiple radiopaque masses suggestive of a compound odontoma associated with the unerupted permanent canine, as well as a horizontally impacted supernumerary tooth (Figure 1a). For further evaluation, a cone beam computed tomography (CBCT) scan was conducted (Figure 1b,c), which confirmed the presence of a cluster of small denticles characteristic of a compound odontoma. The odontoma appeared closely associated with the retained Primary Tooth #73. Table 2 summarizes key clinical and radiographic findings.

Figure 1Pretreatment radiograph. (a) Orthopantomogram (OPG) x‐ray. Orange arrow: impacted 33, aqua arrow: retained 73, red arrow: odontoma, green arrow: supernumerary tooth. (b) Cone beam computed tomography (CBCT). Red arrow: horizontally impacted supernumerary tooth. Green arrow: odontoma. (c) CBCT view of the lingually impacted canine (orange arrow).

**Figure:**
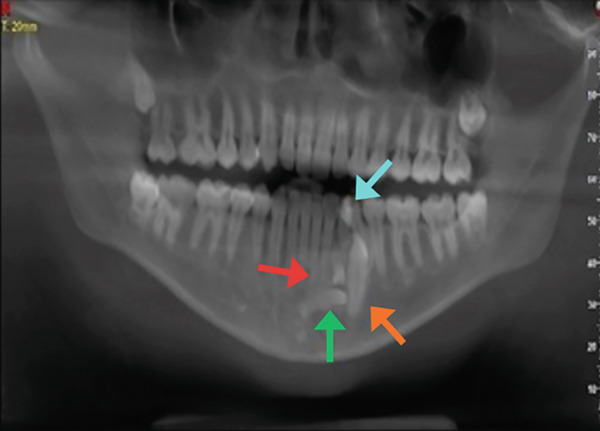
(a)

**Figure:**
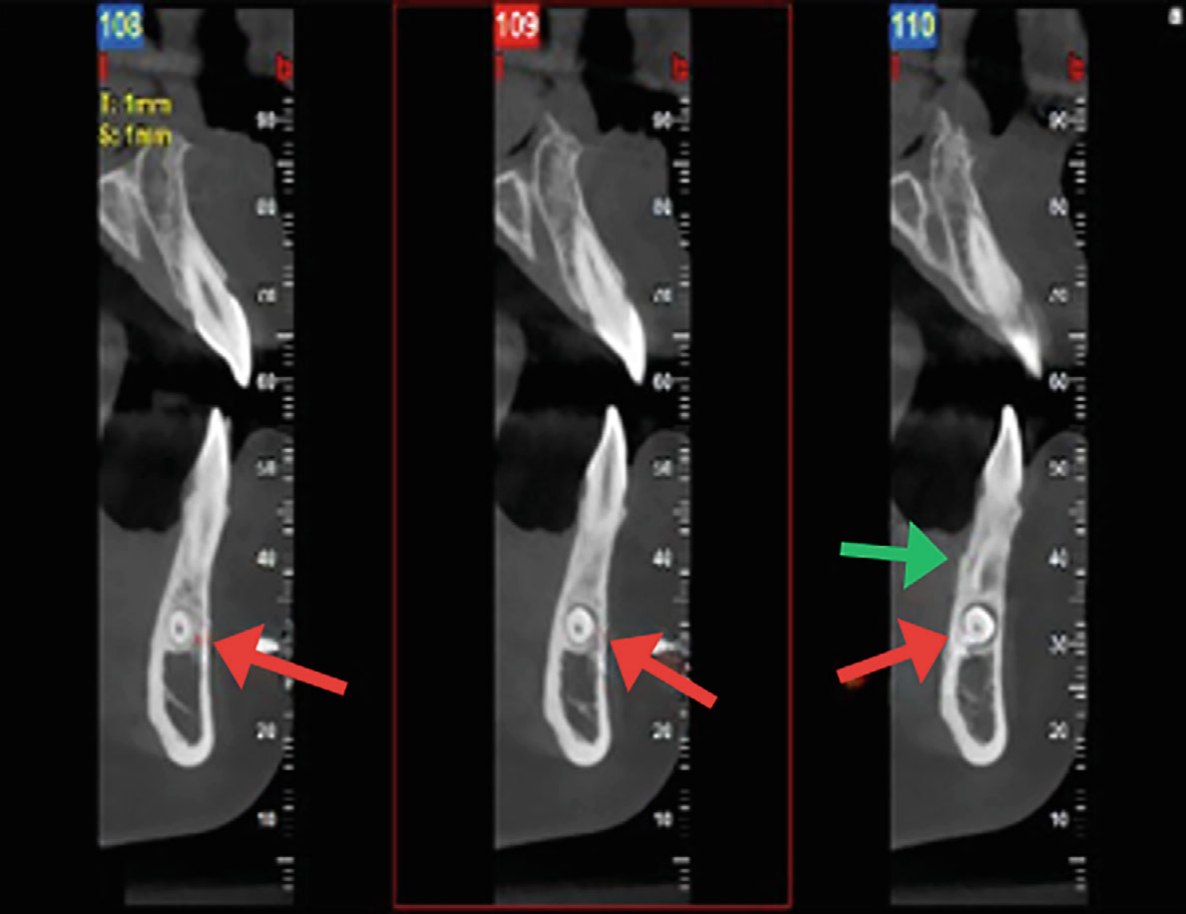
(b)

**Figure:**
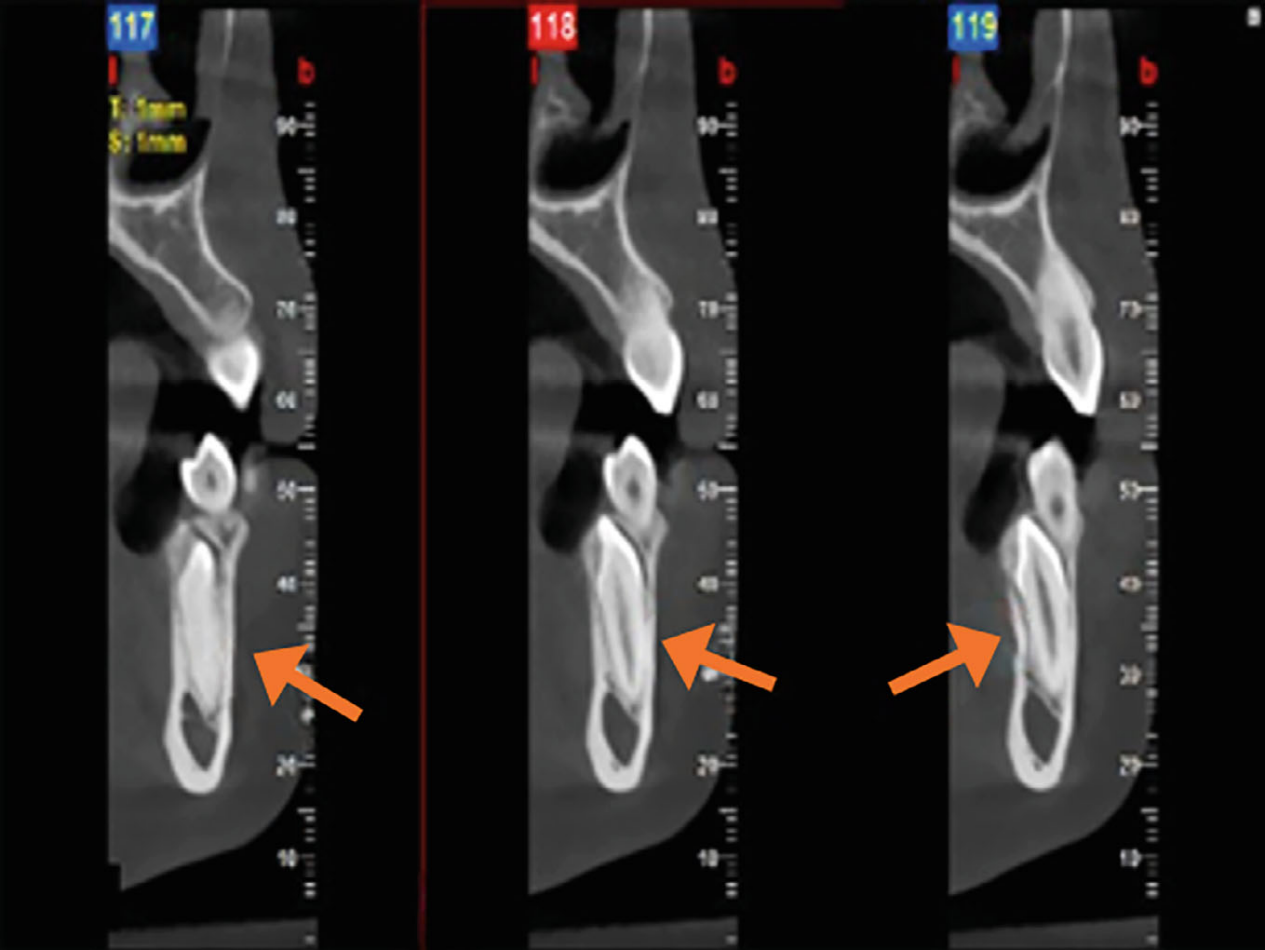
(c)

**Table 2:** Key clinical and radiographic findings.

**Clinical findings**	**Radiographic findings**
Absence of lower left canine #33	Radiopaque masses lower left side
Retained deciduous lower left canine #73	Unerupted lower left canine #33
	Horizontally impacted supernumerary tooth lower left side

The diagnosis, potential implications, and proposed surgical intervention were thoroughly discussed with the patient′s mother. Surgical removal of the lesion was recommended to facilitate eruption of Tooth #33, in conjunction with planned orthodontic treatment. Given the patient′s excellent compliance, the procedure was scheduled to be performed under local anesthesia in a clinical setting. Informed consent for treatment and publication of this case was obtained from the patient′s mother.

### 2.3. Preoperative Stage

To aid in surgical planning, a panoramic radiograph and CBCT scan were obtained to evaluate the morphology, localization, and extent of the compound odontoma, the impacted supernumerary tooth, and the retained primary canine. The imaging revealed a lesion approximately 5 mm in diameter with a radiolucent border surrounding multiple tooth‐like structures (denticles), located apically and distally to the primary canine. Both the patient and her mother were informed in detail about the diagnosis, the nature of the surgical procedure, potential risks, alternative options, and postoperative care.

### 2.4. Operative Stage (Surgical Intervention)

The procedure was carried out in December 2024 under local anesthesia using articaine 4% with 1:200,000 epinephrine (administered as a nerve block). The patient remained cooperative and responsive throughout the procedure without any complications.

**Figure 3:**
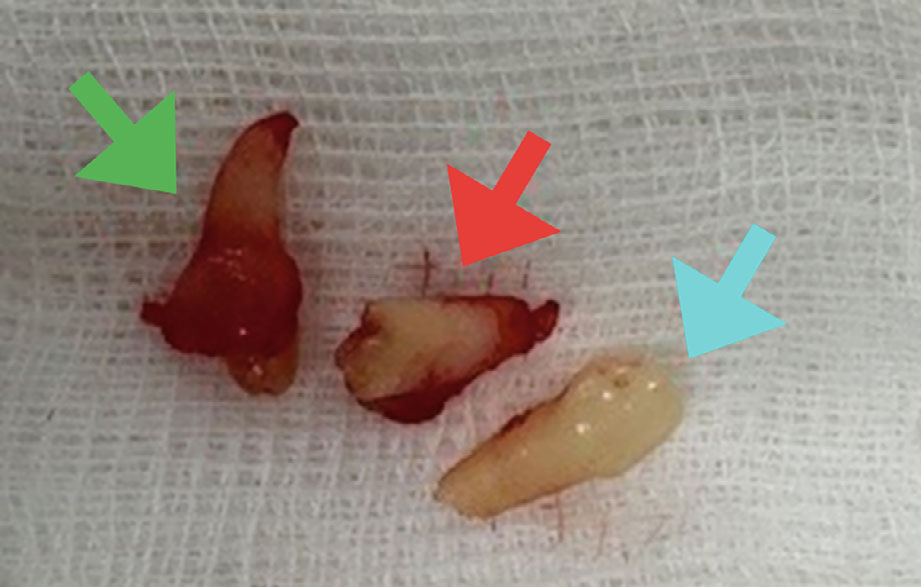
Extracted deciduous canine (aqua arrow), odontoma (red arrow), and supernumerary tooth (green arrow).

The surgical procedure began by removing the deciduous canine (Tooth #73), followed by a marginal and distal releasing incision and dissection of a mucoperiosteal flap to access the underlying bone plane, taking precautions to preserve the mental nerve. The bone cortex appeared partially eroded by neoformation, which was visible immediately below the elevated flap. To gain better access to the lesion, a small portion of the surrounding bone was removed using a surgical bone bur under continuous saline irrigation. This revealed the impacted supernumerary tooth and odontoma (Figure 2a). The odontoma was removed using spade forceps, followed by multiple irrigations and removal of its surrounding connective tissue capsule with an alveolar spoon (Figure 2b). The impacted supernumerary tooth was then removed using the same forceps technique. Curettage of the site was followed by copious saline irrigation, and a collagen sponge was placed into the bony defect for hemostasis and healing support (Figure 2c). The extracted primary canine, odontoma, and supernumerary tooth are shown in Figure 3.

Figure 2(a) Extraction socket of the primary canine (black arrow), visible impacted supernumerary tooth (green arrow) and odontoma (aqua arrow). (b) Removal of the odontoma using the spade forceps (red arrow). (c) The defect was packed with a collagen sponge (black arrow).

**Figure:**
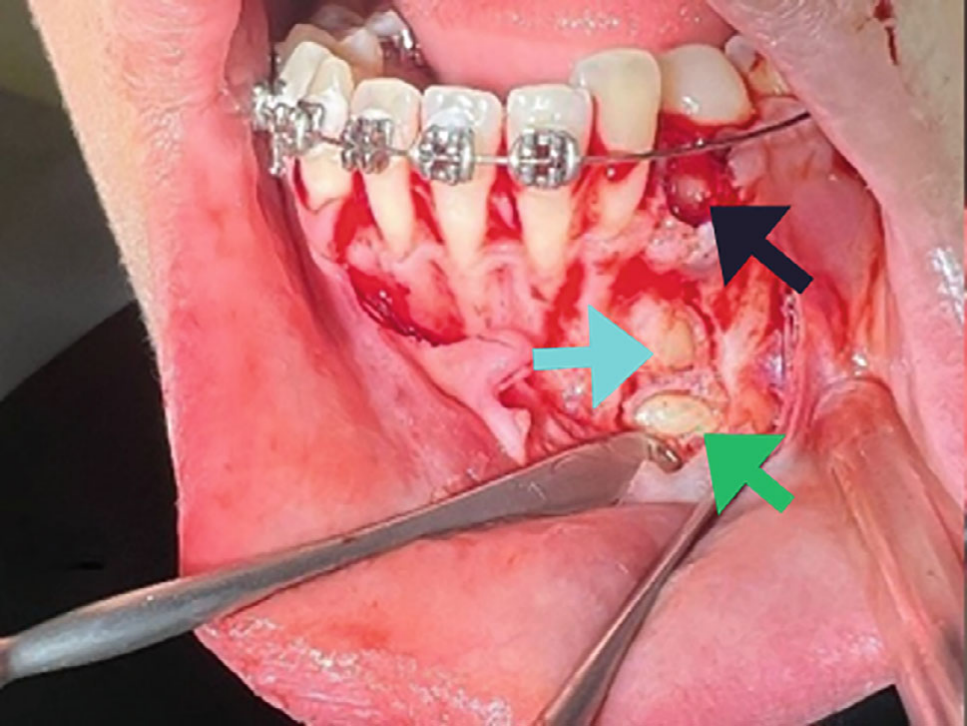
(a)

**Figure:**
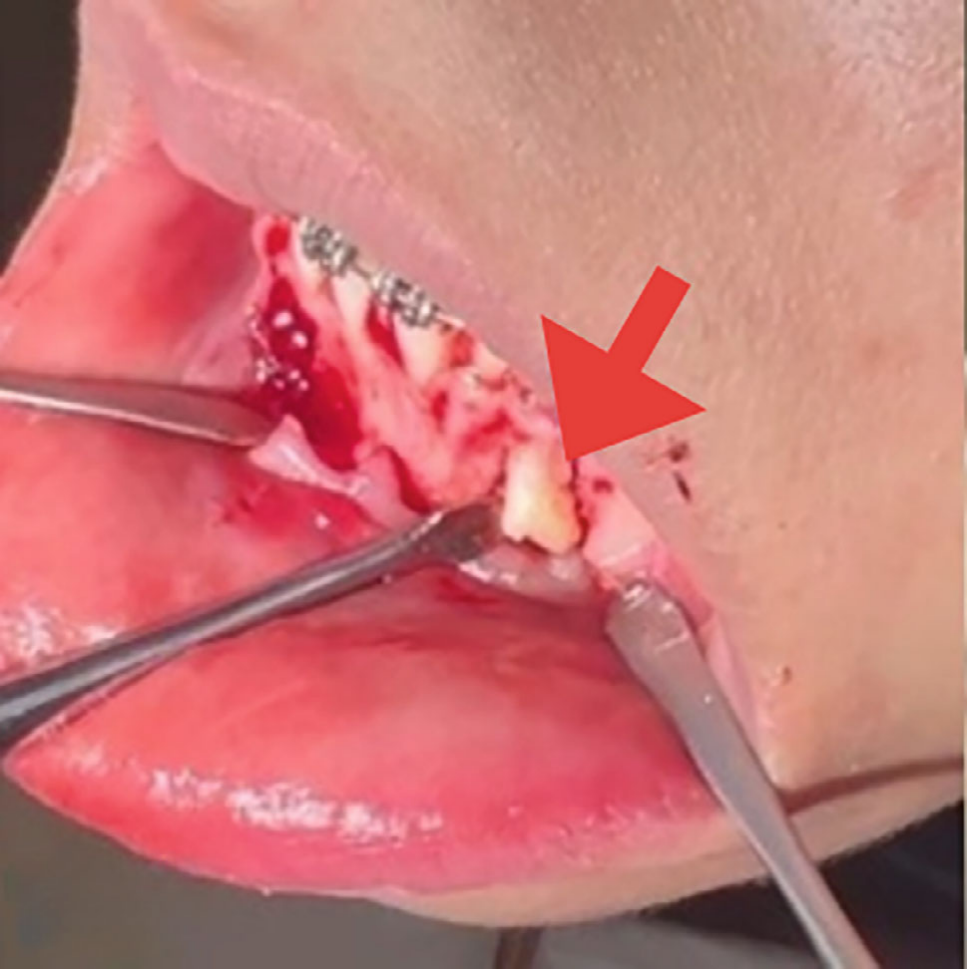
(b)

**Figure:**
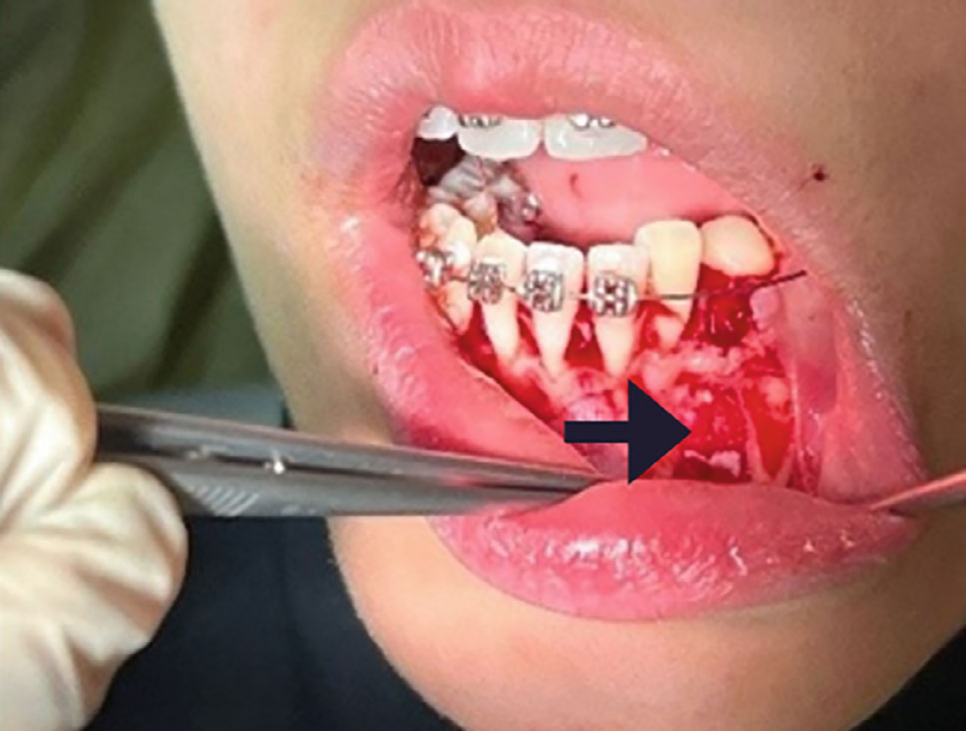
(c)

The lingual plane was approached to surgically expose the impacted permanent canine (Tooth #33). A small marginal incision was made and a mucoperiosteal flap was elevated. Osteotomy was performed under saline irrigation until the crown of the impacted canine was fully visible (Figure 4a), followed by bonding of an orthodontic bracket and chain to the exposed tooth according to the manufacturer′s protocol (Figure 4b). After thoroughly irrigating the surgical site with saline solution, the flap was repositioned and closed with multiple interrupted 4/0 polyglactin 910 sutures (Vicryl Rapide, Ethicon Inc., Somerville, New Jersey, United States) (Figure 4c).

Figure 4(a) Surgical exposure of the impacted canine Tooth 33 (lingual view) (orange arrow). (b) A traction chain was bonded to the lingual aspect of the impacted canine (black arrow). (c) The flap was repositioned and secured with multiple interrupted absorbable sutures (red arrow).

**Figure:**
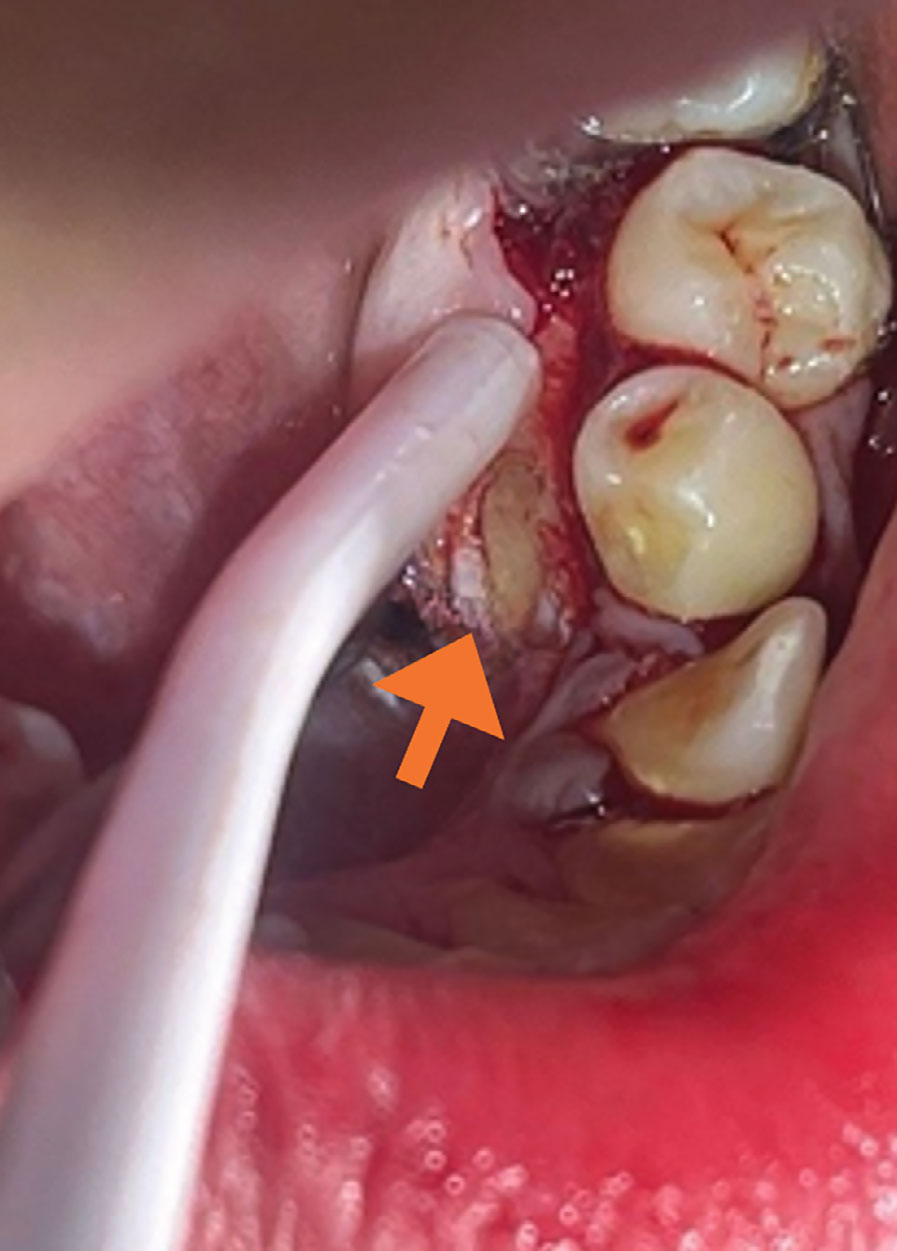
(a)

**Figure:**
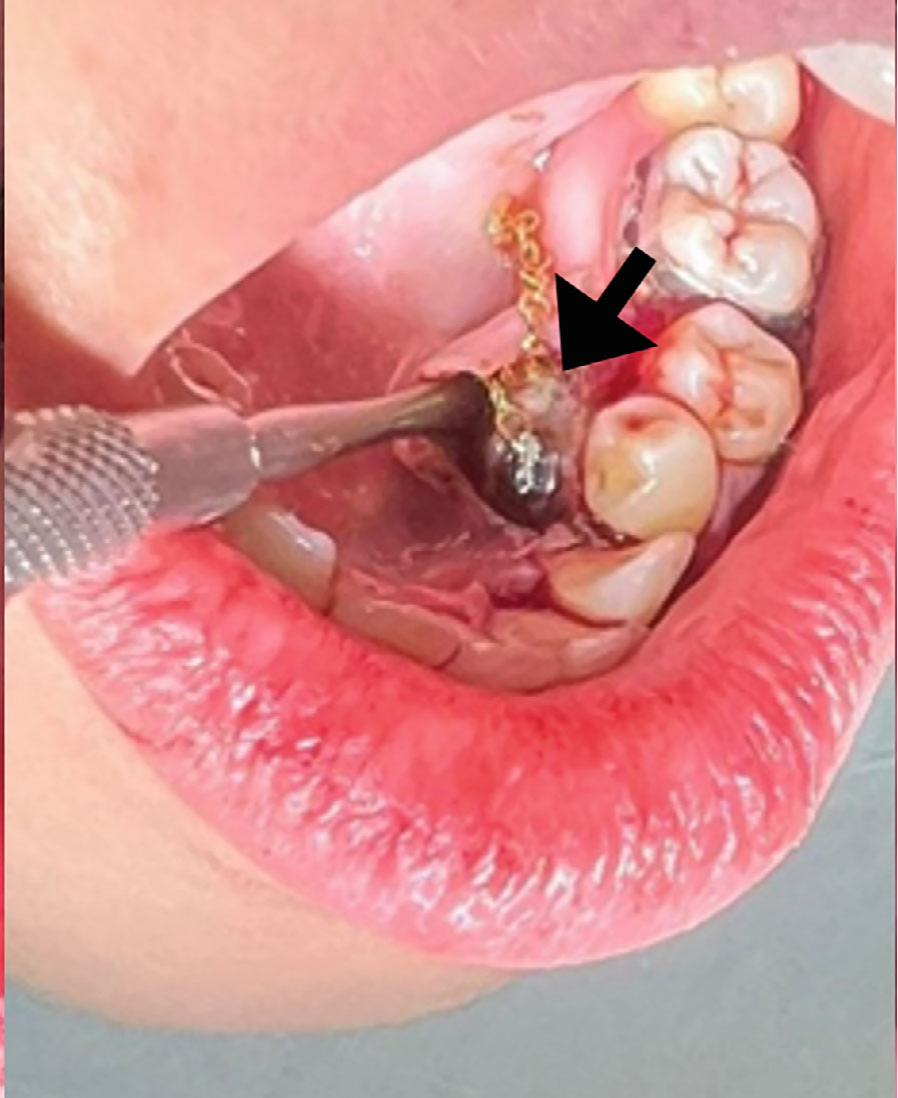
(b)

**Figure:**
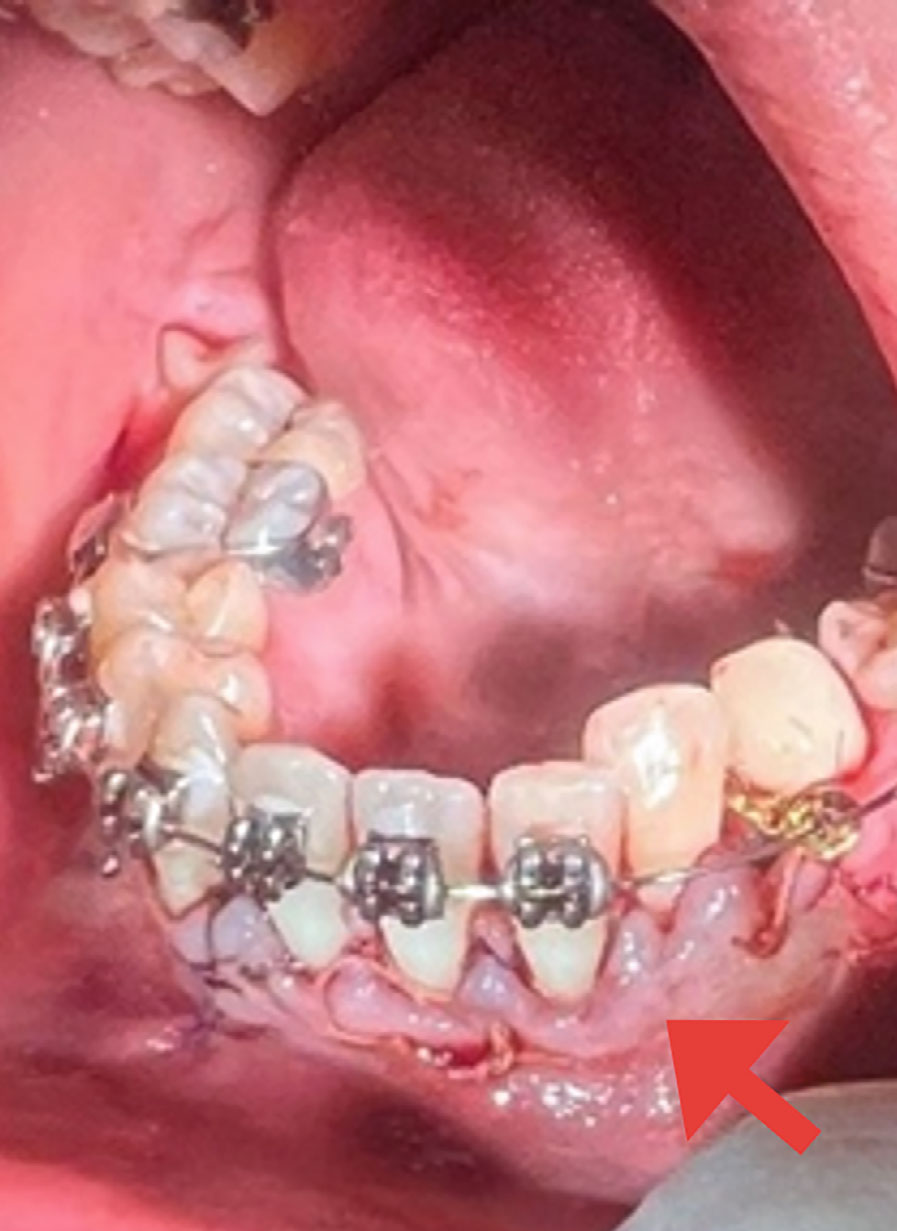
(c)

### 2.5. Postoperative Stage

Postoperative instructions and medications were prescribed, including ibuprofen 400 mg, amoxicillin–clavulanate (Augmentin) 1 g, and vitamin C 500 mg. The patient was scheduled for a follow‐up visit after 14 days to assess soft tissue healing and further orthodontic management by an orthodontist.

### 2.6. Follow‐Ups

**Figure 5:**
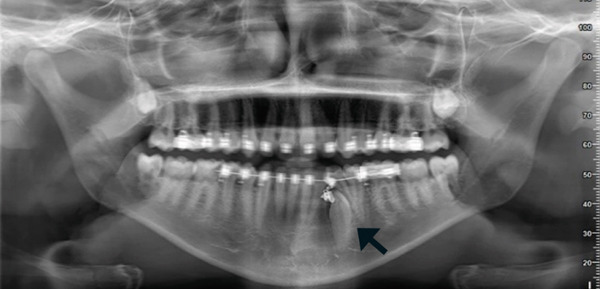
A 2‐week postoperative OPG demonstrates the traction chain affixed to the impacted canine (black arrow).

At the 2‐week follow‐up, the surgical site demonstrated excellent soft tissue healing, with no signs of inflammation except mild swelling and a pain score of 3/10. A new panoramic radiograph (Figure 5) was subsequently obtained for evaluation.

**Figure 6:**
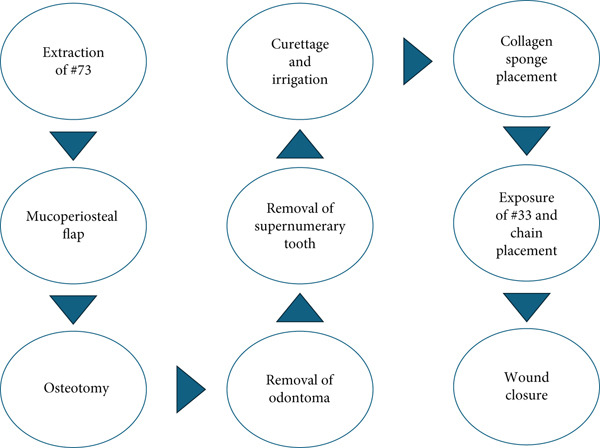
Flowchart outlining the approached surgical procedures.

The 3‐month follow‐up showed no recurrence, and partial eruption of the lower left permanent canine was observed, confirming that the odontoma and retained primary canine had prevented its normal eruptive process. Table 3 and Figure 6 show the timeline of events and the sequence of the interventional procedures.

**Table 3:** Timeline of historical information and events.

**Date**	**Timepoint**	**Key clinical events and findings**	**Investigations/interventions**	**Outcome/comments**
22/5/2024	Presentation (Day 1)	22‐year‐old female; congenital absence of lower left permanent canine (#33); retained deciduous canine (#73); asymptomatic, no swelling/infection	Chair‐side clinical exam	Initial suspicion of odontogenic anomaly
22/5/2024	Initial imaging	—	Panoramic radiograph (OPG) → multiple radiopaque masses; unerupted #33; horizontally impacted supernumerary tooth	Findings suggest compound odontoma obstructing eruption
5/9/2025	Advanced imaging	—	Cone beam CT confirms cluster of denticles (compound odontoma) + supernumerary tooth; proximity to #73	Diagnosis established; surgical removal recommended
3/12/2024	Treatment planning and consent	Case, risks, and alternatives explained to patient and mother	Written informed for surgery and publication	Multidisciplinary plan (surgery + orthodontics) agreed
3/12/2024	Preoperative preparation	—	Additional panoramic/CBCT for surgical mapping; LA protocol discussed	Ready for outpatient procedure
3/12/2024	Surgical intervention	Removal of retained #73; mucoperiosteal flap raised, osteotomy and removal of supernumerary tooth and odontoma, surgical exposure of impacted #33	Enucleation of compound odontoma; extraction of supernumerary tooth; curettage and saline irrigation; collagen sponge placed	Impacted canine (#33) surgically exposed; orthodontic bracket + chain bonded
12/12/2024	Immediate post‐op	—	Wound closure with interrupted Vicryl sutures; meds: Ibuprofen 400 mg, Augmentin 1 g, Vit C 500 mg	Uneventful recovery; follow‐up scheduled
15/12/2024	2‐week follow‐up	No complications; excellent soft tissue healing	Panoramic radiograph obtained	Continues orthodontic care
12/2/2025	3‐month follow‐up	Partial eruption of #33; no lesion recurrence	Clinical/CBCT review	Ongoing traction planned; prognosis favorable
When due	Long‐term follow‐up	Assess full eruption and alignment of #33; watch for ankylosis	Annual imaging	Complete orthodontic outcome/consider prosthetic options if eruption fails

## 3. Discussion

This case presents the occurrence of a compound odontoma in association with several dental anomalies, including an impacted permanent canine, a supernumerary tooth, and a retained deciduous canine. Odontomas are typically diagnosed in response to clinical signs such as failure of eruption of permanent teeth or overretention of deciduous teeth, both signs present in this case [7]. Despite extensive documentation, there is no consensus on the optimal management strategy for impacted teeth associated with odontomas. However, management often necessitates a multidisciplinary surgical approach [8, 9]. In this particular case, the diagnosis and intervention were significantly delayed. The comprehensive imaging‐guided treatment plan, which included excision of the odontoma, extraction of the supernumerary tooth and retained deciduous canine, as well as subsequent orthodontic management, successfully enabled the appropriate alignment of the permanent canine.

Contemporary studies underscore the benefits of early diagnosis and coordinated, multidisciplinary management of odontomas associated with impacted teeth, particularly in pediatric patients, which yield more favorable outcomes [10].

In this case, successful management relied on a multidisciplinary, imaging‐guided approach that combined surgical removal of the odontoma, extraction of the supernumerary and retained deciduous canine, and subsequent orthodontic alignment. This strategy reflects principles emphasized in recent literature, where early collaboration between oral surgeons and orthodontists is considered essential for managing impacted canines associated with odontomas. Reports by Hashim et al. demonstrated that timely intervention—particularly before complete root development—significantly improves treatment outcomes [11]. Similarly, Alhazmi et al. documented favorable results in a 15‐year‐old patient with complex dental anomalies through coordinated surgical and orthodontic care, reinforcing the value of individualized treatment planning [12]. These findings support our decision to integrate advanced imaging and team‐based planning to optimize clinical results.

Evidence from a systematic review further validates this approach, indicating that combined surgical–orthodontic protocols yield high success rates when interventions are timely and tailored to patient needs [13]. Studies on compound odontomas in younger populations consistently highlight that early diagnosis and removal reduce treatment complexity and increase the likelihood of spontaneous or guided eruption [13]. Although our case involved delayed diagnosis and complete root apex closure, orthodontic traction remains justified to achieve functional alignment. Collectively, these insights underscore the importance of early detection and interdisciplinary management to preserve eruptive potential and minimize complications such as ankylosis objectives that should guide future clinical protocols.

In this presented case, at follow‐up, the impacted permanent canine had not yet reached the occlusal plane, raising concerns regarding potential ankylosis, a known complication that can hinder orthodontic repositioning. Additionally, the stage of root development is a critical factor in the eruption process; teeth with closed apices, as in this case, tend to erupt more slowly compared to those with open apices. Considering the patient′s age and delayed diagnosis, the presence of a closed apex may compromise the eruptive potential of the impacted canine. Ongoing monitoring and orthodontic management remain essential. In the event of eruption failure due to ankylosis, surgical extraction of the impacted tooth may become necessary, with subsequent prosthetic rehabilitation through implant placement or a fixed dental prosthesis.

## 4. Conclusion

Odontomas are frequently associated with delayed or failed eruption, often resulting in the impaction of adjacent teeth. Although variations in normal tooth eruption are common, significant deviations from established norms should be further investigated in routine dental check‐ups. Clinical experience and dental literature suggest that an individualized radiographic examination is justified for any pediatric patient showing clinical signs of delayed eruption, irrespective of prior dental trauma or other contributing factors. Early identification enables timely intervention, which is critical to ensuring optimal outcomes. Such procedures may be performed under local anesthesia. However, for noncompliant or anxious pediatric patients, conscious sedation with nitrous oxide or general anesthesia may be indicated to facilitate cooperation and enhance procedural comfort. Ultimately, early diagnosis, supported by a multidisciplinary approach, is essential for initiating timely and appropriate treatment strategies. Such proactive management promotes eruption in alignment with physiological norms, thereby minimizing the risk of developing malocclusions and contributing to improved long‐term oral health.

**Nomenclature**
CBCT: cone beam computed tomography
OPG: orthopantomogram

## Consent

Patient confidentiality was rigorously maintained, and no identifiable information has been revealed. Written informed consent encompassing permission for surgical intervention and to publish the accompanying clinical images and radiographs was obtained.

## Disclosure

All authors have read and approved the final manuscript and agree to be accountable for all aspects of the work.

## Conflicts of Interest

The authors declare no conflicts of interest.

## Funding

The authors declare that no funds, grants, or other forms of financial support were received for the conduct of this study or the preparation of this case report.

## Data Availability

The data that support the findings of this study are available on request from the corresponding author. The data are not publicly available due to privacy or ethical restrictions.
